# Supplementary material for: Genetic linkage mapping and quantitative trait locus (QTL) analysis of sweet basil (Ocimum basilicum L.) to identify genomic regions associated with cold tolerance and major volatiles
Source: PLoS One. 2024 Apr 9;19(4):e0299825. doi: 10.1371/journal.pone.0299825 (PMC11003626; doi:10.1371/journal.pone.0299825)
Supplement: S1 Fig — Flow cytometry for the parents (A-B), hybrid (C), and selected F2 progeny (D-F). Estimated genome sizes of the accessions were 4.85–4.88 Gb (2C DNA content) with support for tetraploid inheritance. (PDF) [file pone.0299825.s002.pdf]

A. CB15

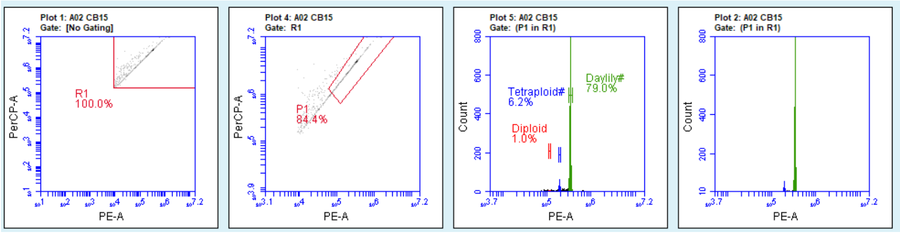


B. Obsession

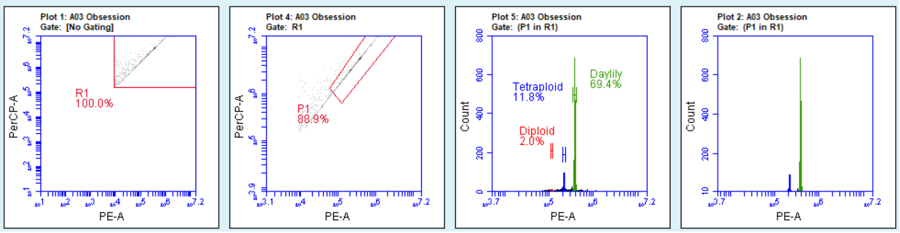


C. F1

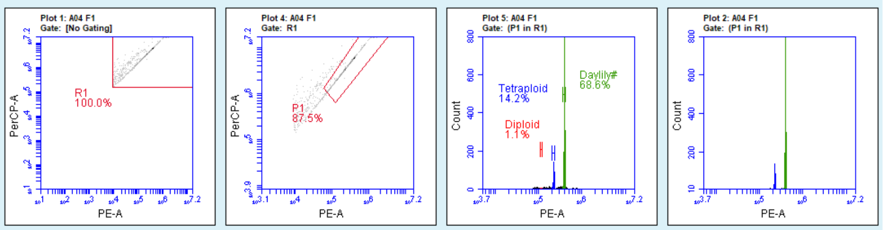


D. OCB-010

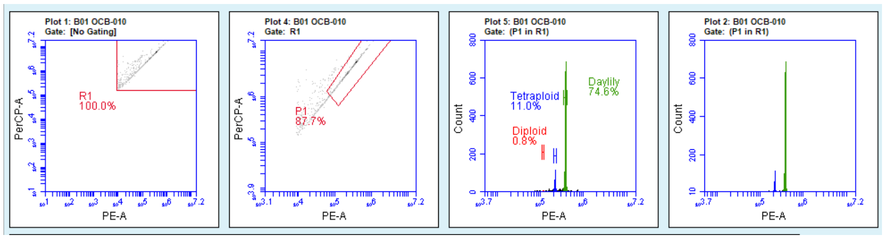


E. OCB-065

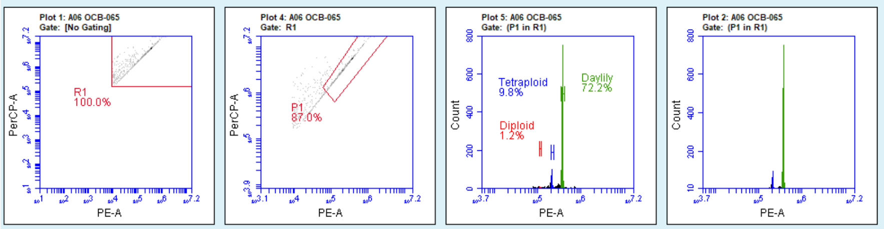


F. OCB-180

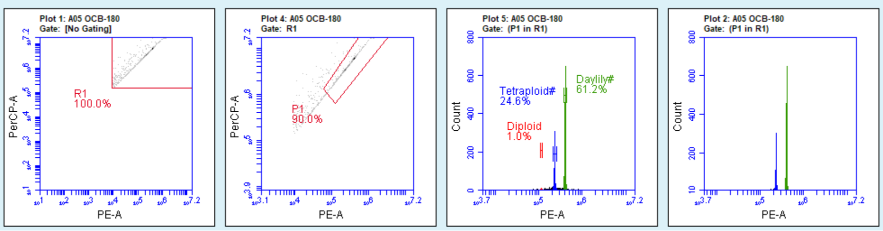


**S1 Figure. Flow cytometry for the parents (A-B), hybrid (C), and select F2 progeny (D-F).** Estimated genome sizes of the accessions were 4.85 – 4.88 Gb (2C DNA content) with support for tetraploid inheritance.
