# Supplementary figures and images for: Genetic linkage mapping and quantitative trait locus (QTL) analysis of sweet basil (Ocimum basilicum L.) to identify genomic regions associated with cold tolerance and major volatiles
Source: PLoS One. 2024 Apr 9;19(4):e0299825. doi: 10.1371/journal.pone.0299825 (PMC11003626; doi:10.1371/journal.pone.0299825)

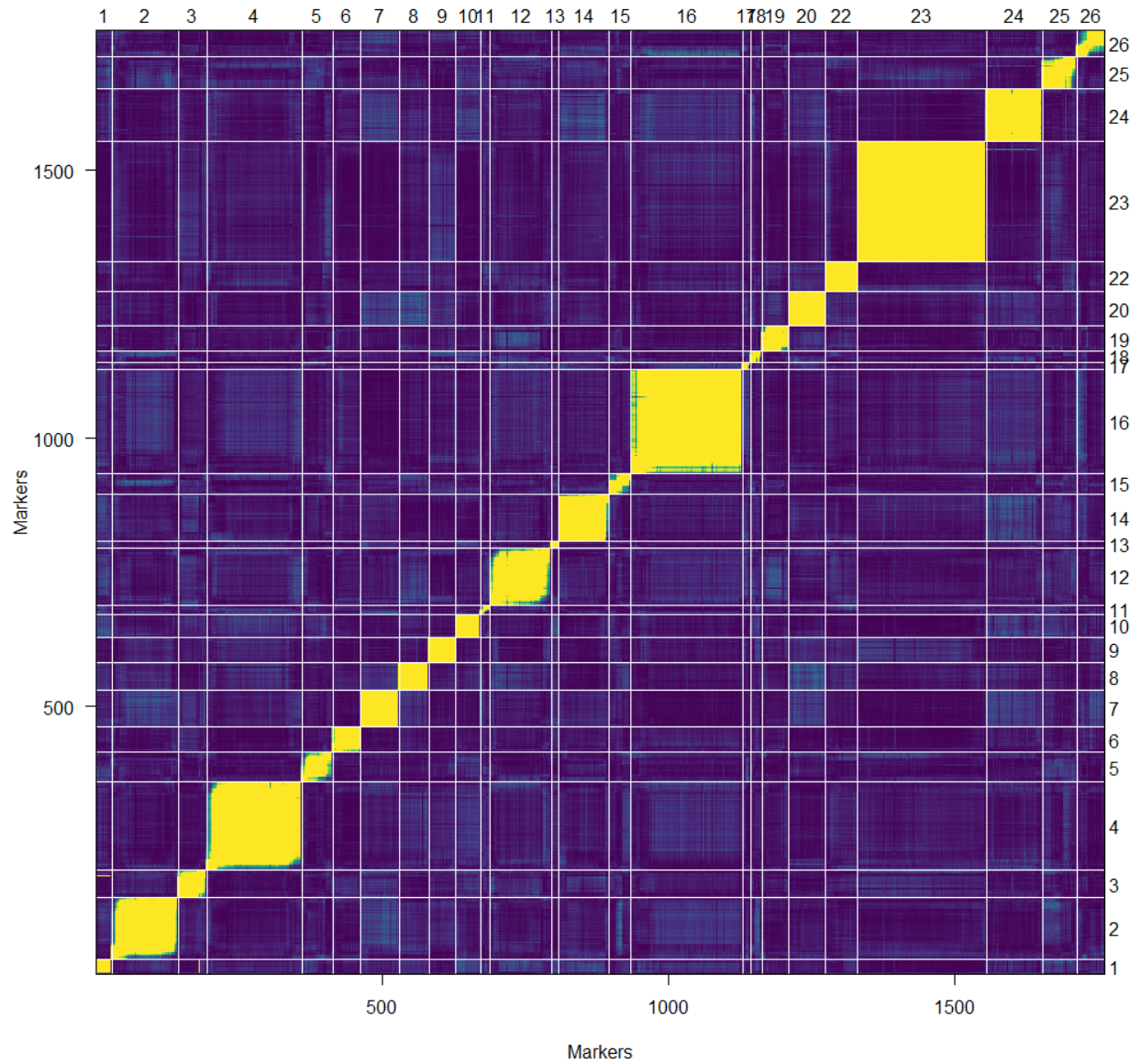

**Figure S2. Heatmap of the marker-pairwise estimated recombination fractions vs LOD scores.**

Supplement: S2 Fig — LOD scores. (PDF) [file pone.0299825.s003.pdf]
